# The correlation between ocular residual astigmatism and wavefront-guided FS-LASIK correction effects in myopic astigmatism patients

**DOI:** 10.1038/s41598-025-08868-5

**Published:** 2025-07-15

**Authors:** Qingyun Zuo, Yu Zhang, Ming He, Siyi Wang, ZhiYu Du

**Affiliations:** 1 Department of Ophthalmology, The Second Affiliated Hospital of Chongqing Medical University, Chongqing, 400010 China; 2 Medal Eye Institute, Chongqing, 400050 China; 3 Department of Ophthalmology, Dazhou Integrated Traditional and Western Medicine Hospital, Dazhou, 635000 China

**Keywords:** Wavefront-guided, FS-LASIK, Ocular residual astigmatism, Vector analysis, Eye diseases, Surgery, Medical research

## Abstract

To evaluate the impact of ocular residual astigmatism (ORA) on the astigmatic correction outcomes of wavefront-guided femtosecond laser-assisted in situ keratomileusis (WFG FS-LASIK) in myopic astigmatism patients. Retrospective cohort study. This study analyzed 90 patients (177 eyes) undergoing WFG FS-LASIK at Chongqing Medal Eye Institute between January 2015 and May 2021, all with complete follow-up data spanning 15 days–6 months postoperatively. Participants were categorized into three groups based on the ratio of ORA to refractive astigmatism (RA), and the axial difference between ORA and anterior corneal astigmatism (ACA): Group1 (42 eyes): |ORA|/|RA| < 1 and the axial difference between ORA and ACA was ≦ 45°. Group 2 (86 eyes): |ORA|/|RA| < 1 and the axial difference between ORA and ACA was > 45°. Group3 (49 eyes): |ORA|/|RA| > 1 and the axial difference between ORA and ACA was > 45°. Uncorrected distance visual acuity (UDVA) at 6 months postoperatively was compared among the three groups. Alpins vector analysis was performed to compare the accuracy of astigmatic correction, quantified by: Angle of error (AE): axial deviation between the surgically induced astigmatism (SIA) and target induced astigmatism (TIA); Correction index (CI): ratio of SIA to TIA (ideal value = 1.0). The mean |AE| values significantly differed among groups (*P* < 0.05), being lowest in Group 1 (9.29°) and highest in Group 3 (26.64°). Correction index (CI) defined as the ratio of SIA to TIA. The mean CI values were1.00 (Group 1), 1.19 (Group 2) and 3.26 (Group 3).No statistically significant differences were observed in postoperative UDVA among the three groups (*P* > 0.05). In WGF FS-LASIK, the consistency between the axis of ORA and ACA affects the degree of deviation in astigmatic axis correction, but had no significant impact on UDVA.

## Introduction

As a significant refractive error, the efficacy of astigmatism correction directly impacts postoperative visual quality. Previous studies demonstrate that uncorrected astigmatism exceeding 0.75D induces characteristic visual disturbances including monocular diplopia, glare, and halo phenomena^1^. The effectiveness of keratorefractive surgery in correcting astigmatism is influenced by various factors. RA derives primarily from ACA, while ORA contributes significantly to RA in certain populations. Keratorefractive surgery corrects astigmatism and other refractive errors by performing ablations on the anterior corneal surface, while ORA represents the non-anterior-corneal component of RA. Studies have found that a higher preoperative ORA may reduce astigmatic correction in SMILE surgery^2^ and LASIK surgery^3,4^. Compared to other keratorefractive surgeries, WFG FS-LASIK surgery has an iris-alignment feature that compensates for cyclotorsion via iris texture, enhancing the precision of astigmatism correction. However, whether ORA similarly impacts its efficacy remains unclear? Regarding the axis, ORA predominantly exerts a partial counteracting effect on ACA, but superposition effects can coexist. Previous research on keratorefractive surgery and ORA have predominantly focused on the magnitude-related effects, whereas the critical role of ORA axis alignment relative to ACA remains underexplored. Therefore, this study intended to use Alpins vector analysis to assess the correlation between the magnitude and axis of preoperative ORA and the effectiveness of astigmatic correction in WFG FS-LASIK surgery.

## Materials and methods

### Participants

We conducted a retrospective cohort study.This study analyzed 90 patients (177 eyes) undergoing WFG FS-LASIK at Chongqing Medal Eye Institute between January 2015 and May 2021, all with complete follow-up data spanning 15 days–6 months postoperatively.There were 39 males (78 eyes) and 51 females (99 eyes), with 90 right eyes and 87 left eyes. The age ranged from 17 to 46 years,with a mean age of 26.21 years (SD:7.65). The inclusion criteria were as follows: Participants exhibited refractive stability (defined as ≤ 0.50 D annual change) for the preceding 2 years. Preoperative spherical equivalent ≤ − 0.50D (hyperopic excluded). Preoperative |RA|≥ 0.25D. Age > 17 years. Discontinuation of soft contact lenses for more than 2 weeks, rigid contact lenses for more than 4 weeks before surgery. No history of ocular diseases or systemic conditions that could affect refractive status, and no history of ocular trauma or surgery. Participants were in good mental health, with no anxiety, depression, or other psychological disorders.

In the fields of mathematics and physics, it is considered that two vectors cancel each other when their angular separation > 90°, otherwise, they augment each other. Given that the axial difference between astigmatism vectors is twice their axial discrepancy, ORA cancel ACA when their axial difference > 45°, Conversely, ORA augments ACA^5^. Participants were categorized into three groups based on the following criteria: Group1 (42 eyes): Predominantly ACA with aligned axes between ACA and ORA. The group was defined by |ORA|/|RA|< 1 and the axial difference between ORA and ACA was ≦ 45°. Group 2 (86 eyes): Predominantly ACA with counteracting axes between ACA and ORA. The group was defined by |ORA|/|RA|< 1 and the axial difference between ORA and ACA is > 45°. Group3 (49 eyes): Predominantly ORA with counteracting axes between ACA and ORA. The group was defined by |ORA|/|RA|> 1 and the axial difference between ORA and ACA was > 45°.

This study was approved by the Ethics Committee of the Second Clinical College of Chongqing Medical University. In accordance with the principles of the Declaration of Helsinki, patients were informed of their inclusion in the clinical study and gave informed consent to participate in the research.

### Methods

Preoperative examinations included: UDVA, corrected distance visual acuity (CDVA), autorefractor (Japan SHIN-NIPPON, ACCUREF-K9001), Slit-lamp biomicroscope, Comprehensive manifest refraction, Wavefront aberration analysis (WaveScan HD aberrometer, AMO, USA), Corneal topography and three-dimensional anterior segment analysis (Sirius system, CSO, Italy), contrast sensitivity (USA VectorVision, CSV-1000E contrast sensitivity meter).

### The calculation of ORA and vector analysis

In this study, all astigmatism values were recorded in minus cylinder format. RA was measured using wavefront aberrometer. ACA was derived from autorefractor, calculated as the dioptric difference between the steepest (K1) and flattest (K2) meridians, with the axis corresponding to the K2 meridian. ORA was determined through vectorial subtraction of ACA from RA. The calculation method was as follows^6^:

$$\begin{gathered} X_{RA} = RA*\cos (2*A_{RA} ),Y_{RA} = RA * \sin (2*A_{RA} ) \hfill \\ X_{ACA} = ACA*\cos (2*A_{ACA} ),Y_{ACA} = ACA*\sin (2*A_{ACA} ) \hfill \\ \left| {ORA} \right| = \left[ {\left( {X_{RA} - X_{ACA} } \right)^{2} + \left( {Y_{RA} - Y_{RA} } \right)^{2} } \right]^{1/2} \hfill \\ \end{gathered}$$

Astigmatism vector analysis was performed using the Alpins vector analysis method, with key parameters including^6–8^ (Table 1):

**Table 1:** Parameters of Alpins vector analysis method.

Parameters	Meaning
Target induced astigmatism vector (TIA)	TIA is the astigmatism vector that needs to be corrected to achieve the desired refractive state. If the target refractive power is 0, TIA is equivalent to the preoperative astigmatism
Surgically induced astigmatism vector (SIA)	SIA is the actual astigmatism vector corrected by the surgery, which equals to the vector difference between the preoperative and postoperative astigmatism
Difference vector (DV)	DV is the vector difference between TIA and SIA, representing the astigmatism that was not corrected by the surgery. The ideal value is 0
Angle of error (AE)	AE is the axial deviation between the SIA and the TIA. The ideal value is 0. A negative AE indicates a clockwise deviation, while a positive AE indicates a counterclockwise deviation
Correction index (CI)	The value is equal to |SIA| / |TIA|, which can be used to adjust the amount of astigmatism correction to achieve full correction. A value greater than 1 indicates overcorrection

*TIA* target induced astigmatism vector, *SIA* surgically induced astigmatism vector, *DV* difference vector, *AE* angle of error, *CI* correction index.

### Surgicl procedure

Preoperative regimen: Administer 0.3% tobramycin eye drops for three days before surgery. Preoperative preparation included instilling 0.5% proparacaine hydrochloride eye drops twice, followed by rinsing the conjunctival sac and disinfecting the surrounding skin. During surgery, drape the surgical area, instilled 0.5% proparacaine hydrochloride eye drops once, taped down the eyelashes, and used a speculum to open the eyelids. Using the IntraLase FS60 femtosecond laser machine (manufactured by Intralase, USA), a corneal flap was created with a hinge located superiorly, preset to a thickness of 100 um and a diameter of 8.5 mm. The corneal flap was lifted to expose the stromal bed. The patient was instructed to fixate on the target light to align with the center of the pupil. The VISX S4 excimer laser (AMO, USA) was then used to ablate the corneal stroma, with a treatment diameter between 6.0 mm and 8.0 mm. After ablation, the stromal bed was rinsed with BSS solution,and the corneal flap was repositioned. Postoperatively, 0.3% tobramycin eye drops and calf blood deproteinized gel were applied. A bandage contact lens was placed, and a rigid transparent eye shield was applied to the operated eye. All surgeries were performed by the same surgeon,with smooth procedures and no complications observed.

### Statistical methods

Statistical analysis was performed using SPSS version 26.0. Quantitative data were expressed as mean ± standard deviation $\left( {{\overline{\text{x}}} \pm {\text{s}}} \right)$, and categorical data were presented as n (%). The Kruskal–Wallis test (H test) was used to compare preoperative and postoperative parameters among the three groups. A *p*-value of less than 0.05 was considered statistically significant. For significant results, pairwise comparisons were further conducted using the Bonferroni correction, with an adjusted *p*-value threshold of 0.05. For data conforming to a normal distribution, Pearson correlation analysis was used, while Spearman correlation analysis was employed for non-normally distributed data.

## Results

### Preoperative patient demographics and postoperative vector analysis results

**Fig. 1:**
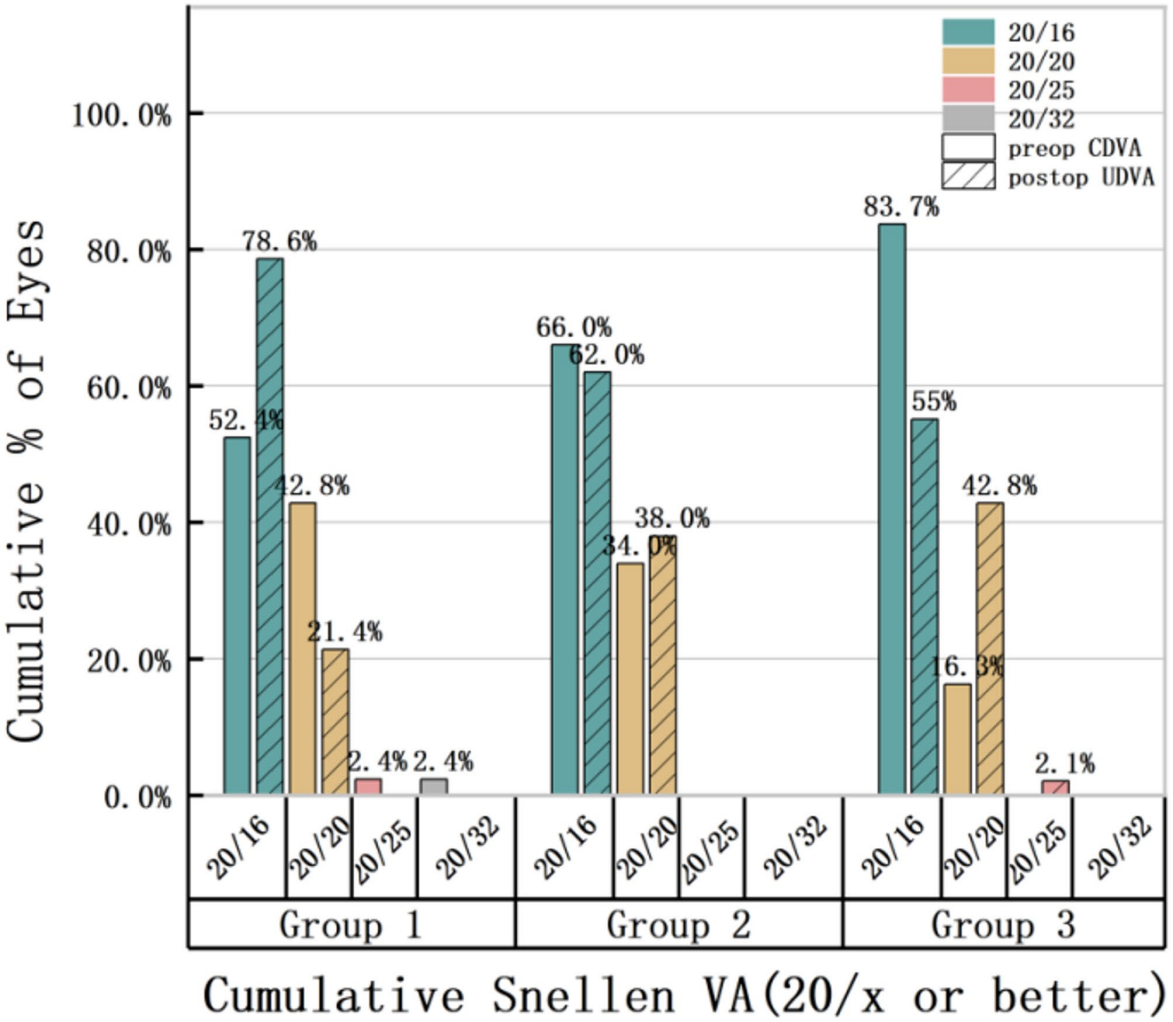
Comparation of cumulative Snellen Visual Acuity among groups.

**Fig. 2:**
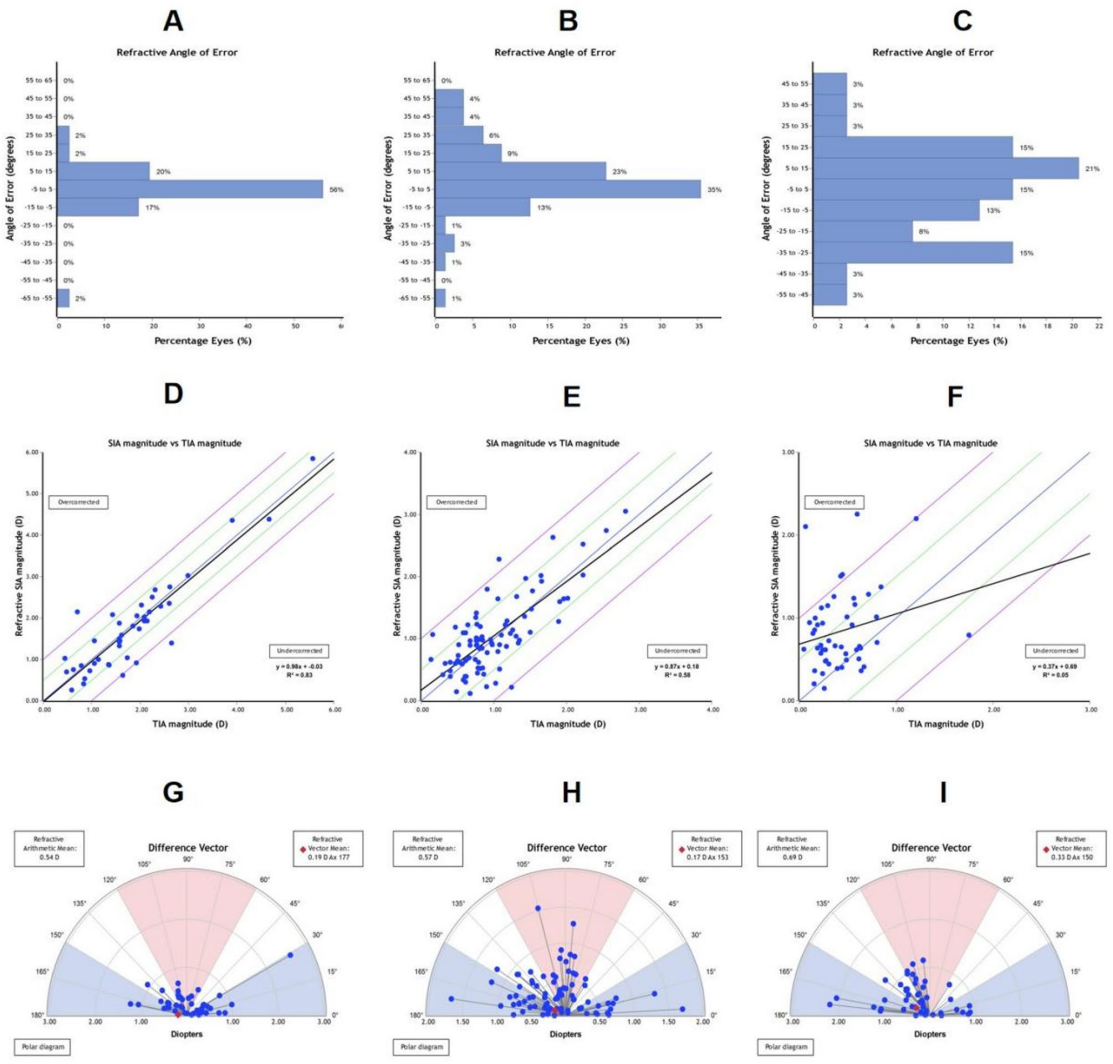
(**A**–**C**) angle of error histogram on which the negative values indicate that the SIA is clockwise to the TIA and positive values indicate that the SIA is counterclockwise to the TIA of Group1, Group2, Group3. (**D**–**F**) linear regression for the prediction of surgically induced astigmatism vector (SIA) by means of the target induced astigmatism vector (TIA) of Group1, Group2, Group3. (**G**–**I**) single-angle polar plots for the difference vector of Group1, Group2, Group3.

Table 2 presents the general preoperative information and the postoperative vector analysis outcomes. No statistically significant intergroup differences were observed in preoperative parameters including age, UDVA and sphere (all *p* > 0.05). Postoperative 6-month UDVA outcomes (mean ± SD): − 0.05 ± 0.05 logMAR (Group 1), − 0.04 ± 0.04 logMAR (Group 2), − 0.04 ± 0.04 logMAR (Group 3), with no statistically significant intergroup differences (*P* > 0.05). Figure 1 demonstrates postoperative UDVA outcomes exceeding 20/25 in all groups, with 20/20 attainment rates of 100% (Group 1), 100% (Group 2), and 97.8% (Group 3), compared to preoperative CDVA distributions. The SIA and TIA values for the three groups showed a sequential decrease with statistically significant differences (*P* < 0.05). There were no significant statistically overcorrection or undercorrection in Group 1 and Group 2 (Fig. 2D,E), while Group 3 showed clinically significant overcorrection (Fig. 2F). Furthermore, Group 1 and Group 2 achieved ideal CI values (1.00 ± 0.46 and 1.19 ± 0.87, respectively), while Group 3 showed obvious overcorrection (3.26 ± 4.93). Statistical differences were observed only between Group 1 and Group 3 (*P* < 0.05), between Group 2 and Group 3 (*P* < 0.05). There were no statistically significant differences in DV (D) or DV (°) among three groups (*P* < 0.05). The mean |AE| value was minimal in Group 1 (9.19 ± 16.02)°, and maximal in Group 3(26.64 ± 21.88)°. All intergroup comparisons reached statistical significance (*P* < 0.05). 91% eyes of |AE| value were within 15° (Fig. 2A) in Group 1, 71% eyes of |AE| value were within 15° (Fig. 2B) in Group 2,while in Group 3, only accounting for 38% (Fig. 2C). The DV of three groups were depicted in single-angle polar plots (Fig. 2G–I).

**Table 2:** Preoperative characteristics and postoperative vector analysis.

Variable	Group 1	Group 2	Group 3	*P*-value
Number of eyes	42	86	49	–
Preoperatively				
Age (years)	25.04 ± 7.48	26.27 ± 7.28	27.29 ± 6.47	0.212
UDVA (LogMAR)	1.17 ± 0.39	1.12 ± 0.36	1.13 ± 0.25	0.775
CDVA (LogMAR)	− 0.03 ± 0.06	− 0.05 ± 0.03	− 0.07 ± 0.02	0.004*
Spherical equivalent (D)	− 7.07 ± 2.87	− 5.51 ± 2.38	− 5.44 ± 2.17	0.038*
Sphere (D)	− 6.06 ± 2.92	− 4.93 ± 2.31	− 5.20 ± 2.17	0.324
RA(D)	− 2.11 ± 1.12	− 1.13 ± 0.61	− 0.49 ± 0.34	< 0.001*
ACA(D)	− 1.79 ± 1.06	− 1.36 ± 0.75	− 0.74 ± 0.48	< 0.001*
|ORA|(D)	0.43 ± 0.22	0.46 ± 0.25	0.92 ± 0.75	< 0.001*
6 months postoperatively				
UDVA (LogMAR)	− 0.05 ± 0.05	− 0.04 ± 0.04	− 0.04 ± 0.04	0.166
RA(D)	− 0.54 ± 0.43	− 0.57 ± 0.35	− 0.70 ± 0.45	0.071
ACA(D)	− 0.89 ± 0.48	− 0.64 ± 0.37	− 0.84 ± 0.53	0.011*
|ORA|(D)	0.72 ± 0.0.39	0.66 ± 0.33	0.72 ± 0.45	0.855
TIA(D)	1.82 ± 1.05	0.99 ± 0.53	0.43 ± 0.30	< 0.001*
SIA(D)	1.76 ± 1.13	1.04 ± 0.61	0.84 ± 0.48	< 0.001*
DV(D)	0.54 ± 0.43	0.57 ± 0.34	0.69 ± 0.44	0.068
DV(°)	83.21 ± 58.74	103.83 ± 55.87	110.69 ± 57.66	0.093
|AE|(°)	9.19 ± 16.02	17.71 ± 20.26	26.64 ± 21.88	< 0.001*
CI	1.00 ± 0.46	1.19 ± 0.87	3.26 ± 4.93	< 0.001*

*UDVA* uncorrected distance visual acuity, *CDVA* corrected distance visual acuity. *D* diopter, *TIA* target induced astigmatism, *SIA* surgically induced astigmatism, *DV* difference vector, *AE* angle of error. *CI* correction index. Kruskal–Wallis test was used for the three-group comparison, followed by Bonferroni-adjusted pairwise comparisons. * significantly different among groups. *P*-value of < 0.05 was considered statistically significant.

### Preoperative and postoperative axis and magnitude distribution and correlations of RA, ACA, and ORA

**Fig. 3:**
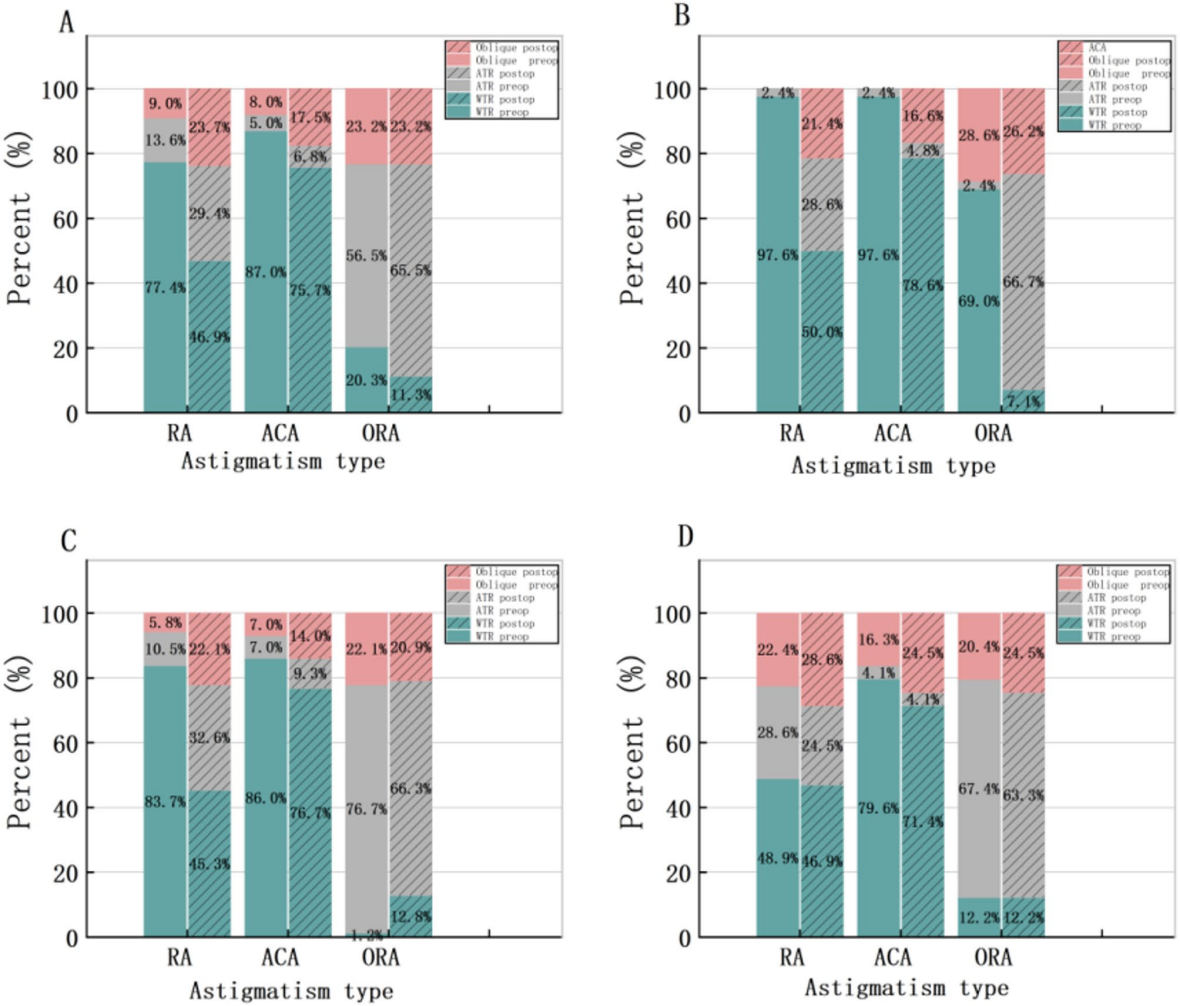
Distributions of the axis of RA, ACA, and ORA by group before and after surgery. A. Total sample. B. Group1. C. Group2. D. Group3. ORA were mainly ATR while ACA and RA were mainly WTR except Group2 before and after surgery. *RA* refractive astigmatism, *ACA* anterior corneal astigmatism. *ORA* ocular residual astigmatism, *WTR* with-the-rule, *ATR* against-the-rule.

**Fig. 4:**
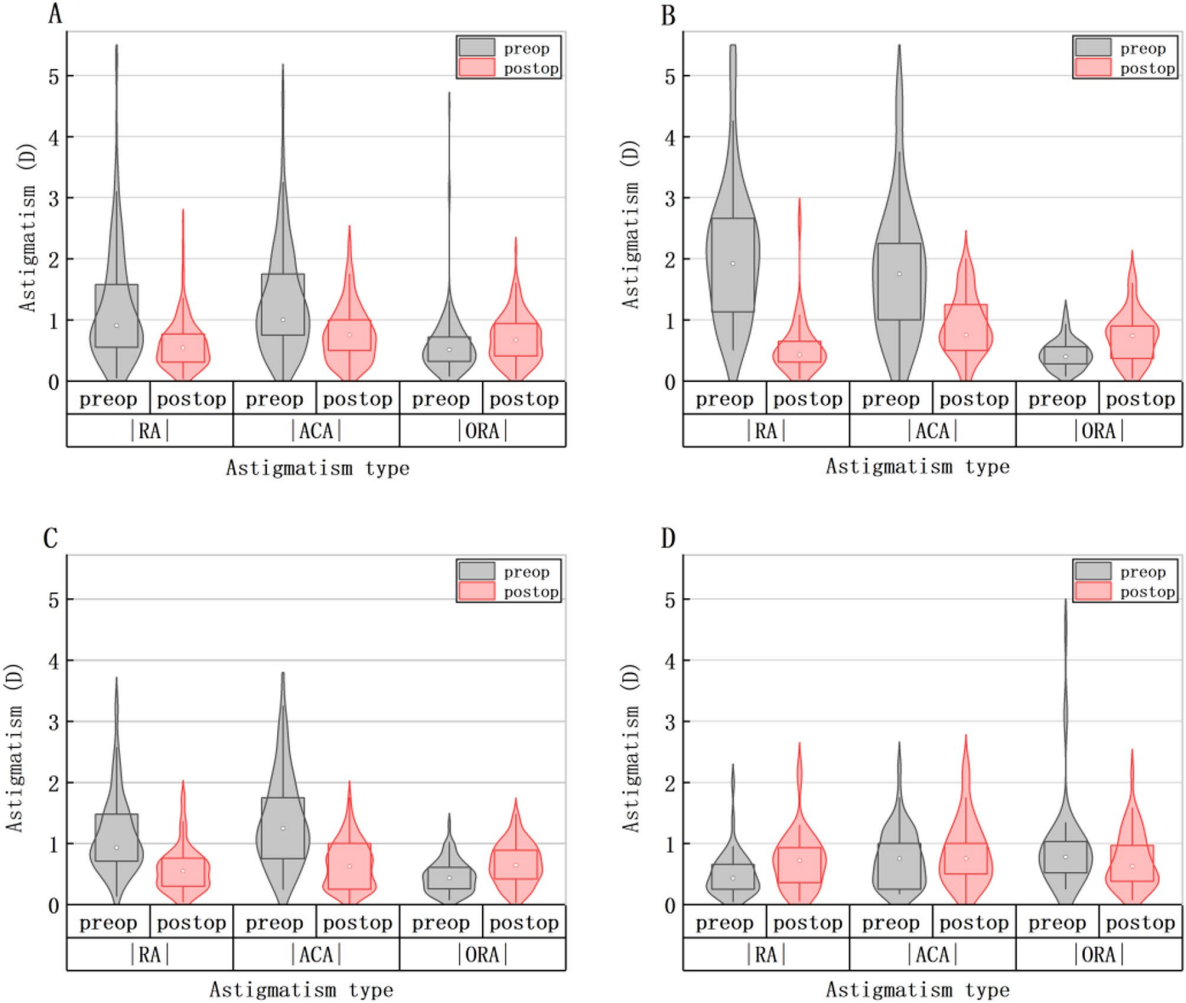
Distributions of the maginitude of RA, ACA, and ORA by group before and after surgery. (**A**) ACA and ORA. (**B**) RA and ORA. (**C**) ACA and ORA. D = Dioptor. preop = preoperative. postop = postoperative. In each plot,The width of the violin plot represents the density of the corresponding data.The rectangle represents the quartile range, the upper and lower tentacles represent the distribution of the data, and the white dots inside represent the median.

In this study, astigmatism was classified according to axis orientation with a 30°threshold. Against-the-rule (ATR): steep meridian between 60°and 120°. With-the-rule (WTR): steep meridian between 0°and 30°, between 150°and 180°. Oblique: all other axis orientations. Figure 3 demonstrates the distributions of the axis of RA, ACA, and ORA. In the total sample, preoperative RA and ACA were primarily WTR, accounting for 137(77.4%), 154(87.0%) respectively, while ORA was primarily ATR as 100(56.5%). The proportion of WTR in RA and ACA decreased postoperatively, but WTR remained predominant. A similar trend was observed in Groups 2 and 3. In Group 1, ORA were primarily ATR as 29 (69.0%) preoperatively, ATR became predominant postoperatively,as 28(66.7%). In our study, in the total sample, the RA, ACA, ORA were (− 1.18 ± 0.92) D, (− 1.29 ± 0.86) D, (− 0.58 ± 0.50) D preoperatively, (− 0.60 ± 0.40) D, (− 1.31 ± 0.59) D and (− 0.69 ± 0.38) D postoperatively. In Group 1, the RA, ACA were (− 2.11 ± 1.12) D, (− 1.79 ± 1.06) D preoperatively, were (− 0.54 ± 0.43) D and (− 0.89 ± 0.48)D postoperatively. In Group 2, the RA, ACA were (− 1.13 ± 0.61) D and (− 1.36 ± 0.75) D preoperatively, were (− 0.57 ± 0.35) D and (− 0.64 ± 0.37) D postoperatively. In Group 3, the RA, ACA were (− 0.49 ± 0.34) D and (− 0.74 ± 0.48) D preoperatively, were (− 0.70 ± 0.45) D and (-0.84 ± 0.53) D postoperatively. |ORA| increased postoperatively in all groups except Group 3. The Wilcoxon signed-rank tests confirmed significant postoperative changes in all astigmatism components (*P* < 0.001). Figure 4 shows the distribution of the magnitude of RA, ACA, and ORA by group before and after surgery, including data distribution density, quartiles, maximum and minimum values, and median. In each violin plot, the width of the violin indicates the data distribution density, the top and bottom edges of the rectangle represents the quartiles, the endpoints of the vertical line represents the maximum and minimum values, and the white dot denotes the median. Considering the data distribution density and range, we can see that the magnitude of both |RA| and |ACA| decreased postoperatively, while |ORA| increased in the total sample, Group 1 and Group 2.

**Fig. 5:**
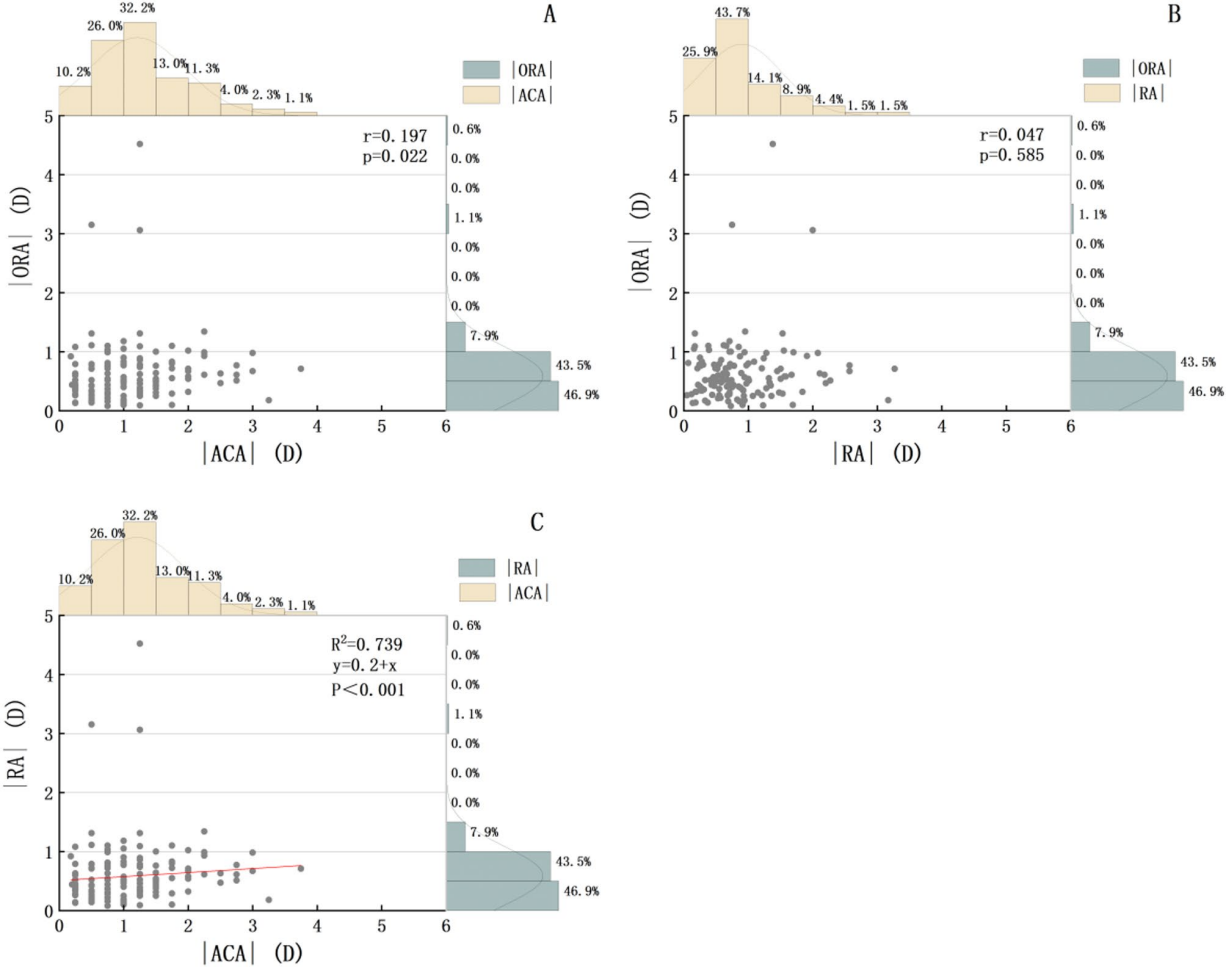
Correlation and disitrbution between the magnitude of RA, ACA and ORA before surgery. (**A**) A weak correlation between ACA and ORA. (**B**) No correlation between RA and ORA. (**C**) A strong correlation between ACA and ORA. D = Dioptor.

Figure 5 illustrates the preoperative distributions and correlation analyses of magnitude values for RA, ACA and ORA. The prevalence of ≤ 1.0 D astigmatism with |ORA| was 90.4%,while the prevalences for ≤ 2.0 D astigmatism with RA and ACA were 81.4%, 92.6% respectively. Spearman correlation analysis was performed to assess the relationships between ACA, ORA, and RA using absolute values of all astigmatism parameters. The analysis revealed: A weak correlation between |ACA| and |ORA|| (r = 0.197, *p* = 0.022), no correlation between |RA| and |ORA| (r = 0.047, *p* = 0.585), a strong correlation between |RA| and |ACA| (R2 = 0.778, *p* < 0.001). Further performing regression analysis to construct a regression equation in |RA| and |ACA| (y = 0.2 + x), indicating that |RA| and |ACA| have a linear relationship.

## Discussion

To assess the correlation between preoperative ORA and the astigmatic correction effect of WFG FS-LASIK surgery, 177 eyes included.The subjects were divided into three groups:Group1(42 eyes): ACA-dominant eyes with aligned axis between ACA and ORA.Group2(86 eyes): ACA-dominant eyes with counteracting axis between ACA and ORA. Group3 (49 eyes): ORA-dominant eyes with counteracting axis between ACA and ORA. The comparison of the results between Group 1 and Group 2 reflected the correlation between the axis of ORA and the effect of surgery,while the comparison of the results between Group 1 and Group 3 reflected the impact of the magnitude of ORA.

Our study used Alpins vector analysis to calculate the AE and CI values for each group, thereby quantitatively assessing both magnitude and axis deviations in astigmatic correction. By analyzing postoperative UDVA and residual astigmatism for three groups, we assessed whether ORA affects the postoperative correction outcome. We also analyze the distribution characteristics of the different astigmatism components.

In vector analysis, the CI value is used to assess the the deviation of magnitude of RA in astigmatic correction. The CI value is numerically equal to the ratio of |SIA| to |TIA|. The ideal CI value is equal to 1, with a CI value greater than 1 indicating overcorrection by the surgery, while less than 1 indicating under correction. In our results, we found that the CI values for both Group 1 and Group 2 were close to 1, with no statistically significant difference between the two groups. These results demonstrate that when preoperative RA is predominantly attributable to ACA, WFG FS-LASIK surgery achieves near-ideal correction, with ORA interaction effects being clinically negligible. Group 3 demonstrated significant overcorrection. To identify the reasons, we further explored and found that the data in Group 3 met the condition where preoperative |ORA|/|RA|> 1 and ORA partially offseted ACA. As a result, this cohort exhibited the highest preoperative ORA magnitude alongside the lowest RA, resulting in ORA neutralizing a substantial portion of ACA. For this reason, we speculate that the overcorrection observed in Group 3 is related to the low preoperative RA. The surgery was designed based on preoperative RA, and the astigmatism introduced by factors such as the creation of the corneal flap, the healing process of the corneal flap, poor cooperation of the eye during surgery, and decentration of the ablation may have masked the actual amount of astigmatism corrected by the surgical design. Consequently, the overall postoperative effect demonstrated overcorrection, with newly induced astigmatism.The conclusion is consistent with previous research. Katz et al.^9^ included 153 eyes and grouped them based on the magnitude of preoperative RA, Using the Alpins vector analysis method, found that wavefront-optimized LASIK surgery tended to overcorrect for low myopic astigmatism (≤ 0.5D).Frings et al.^10^ further expanded the sample size (n = 448 eyes) and reached the same conclusion.

However, CI exclusively quantifies the magnitude ratio of TIA and SIA, without considering deviations of axis. Alpins^11^ believes that an axial deviation of 15° will reduce the correction effect by 13%, and an axial deviation of 30° will halve the correction effect. To address this, we further calculated the AE. AE represents the axial difference between SIA and TIA. A negative AE value indicates that SIA is located clockwise relative to TIA, while a positive value indicates a counterclockwise direction. We used the the absolute value of AE (|AE|) for statistical analysis, with an ideal |AE| being 0°. We found that Group 1 demonstrated the lowest, followed by Group 2. The proportion of eyes with |AE| values within 25° was 95% in Group 1 and 81% in Group 2. Group 3 had the largest |AE| value with a more dispersed distribution, and only 72% of the |AE| values were within 25°.This study suggests that when preoperative RA is predominantly attributable to ACA (especially with aligned axis between ACA and ORA), WFG FS-LASIK results in smaller axial deviations. Optical physics dictates that two astigmatic vectors of equal magnitude but orthogonal axes will completely cancel each other. Consequently, when planning surgical correction for a given astigmatic vector, modifying only the treatment axis while maintaining constant magnitude inherently produces variable residual astigmatism due to vector summation principles. Therefore, we further speculate that the increase in postoperative RA observed in Group 3 may be related not only to surgically induced astigmatism but also to axial rotation during the procedure.

From the results above, it can be seen that in Group 3, the precision of surgical correction in magnitude and axis of preoperative RA was inferior to that the other two groups. Does this mean the postoperative outcome for this group is poor? We further conducted a statistical analysis of the preoperative CDVA and postoperative UDVA for each group. The preoperative CDVA (LogMAR) were as follows: − 0.03 ± 0.06(Group 1), − 0.05 ± 0.03(Group 2), − 0.07 ± 0.02(Group 1). Postoperative UDVA for all three groups (expressed in Snellen scores) was 20/22 or better. Group 3 achieved optimal visual outcomes with both preoperative CDVA and postoperative UDVA. We hypothesize that: The lowest preoperative RA in Group 3 contributed to its superior preoperative CDVA. Despite slight surgically induced astigmatism and minimal axis deviation occurred, the low magnitude of these changes prevented clinically significant degradation in postoperative refraction. These outcomes are likely attributable to the WGF-LASIK treatment algorithm, which customizes ablation patterns based on total ocular wave front aberrations. Furthermore, our prior research on preoperative ORA demonstrated comparable postoperative visual quality outcomes regardless of astigmatism dominance patterns^12^.

Our study also analyzed the distribution patterns of astigmatic components. Regarding axial distribution, preoperative RA and ACA were predominantly exhibited WTR, consistent with eyelid pressure-induced corneal flattening along the horizontal meridian. Since ACA is the primary component of RA, most people tend to exhibit WTR. ORA showed ATR dominance, suggesting its compensatory role in partially neutralizing ACA’s WTR tendency. These findings are consistent with previous studies^13^. Regarding characteristics of magnitude, prevalences for ≤ 2.0D astigmatism with ACA and RA were 81.4%, 92.6%.prevalences for ≤ 1.0D astigmatism with ORA was 90.4%.The average preoperative |ORA| was 0.58D.Mehrdad et al.^14^ analyzed data from 188 myopic individuals (aged from 20.0 to 52.0 years old) and found an average |ORA| of 0.5D. Li et al.^15^ reported an average |ORA| value of 0.2D from 173 Chinese children (aged from 10.0 to 15.6 years old), while Frings^16^ reported an average ORA of 0.5D from 291 eyes (aged from 18.0 to 71.0 years old). The observed outcomes may be influenced by interstudy variations in baseline characteristics, including age, myopia, and astigmatism levels. However, the |ORA| values in all four studies were similar, within 1D, which is consistent with other previous studies^17,18^. Additionally, by comparing the preoperative and postoperative ORA, we found that ORA is not constant. Previously, we considered ORA to be a component of non-corneal anterior surface astigmatism, primarily originating from the posterior corneal surface, the lens, the vitreous body, or even the retina^19,20^.Since keratorefractive surgery is performed only on the corneal anterior surface, the surgery does not directly cause changes in ORA.Therefore, we speculate that the changes in ORA postoperatively might be related to its indirect calculation, meaning that ORA could change in response to variations in RA or ACA. However, in our study, the magnitude of ORA showed no correlation with RA and only a weak correlation with ACA. Based on this, we speculate that the magnitude of ORA may not be a constant value. ORA may be not only an optical concept, changes in refractive status could affect the subjective perception of the human eye and further influence the complex neural processing of astigmatism. The notion of whether ORA is a constant value is rarely reported, and the correlation between ORA and ACA or RA remains controversial. These may related multiple dynamic factors, including: Postoperative refractive status, Pupillary dynamics, Crystalline lens accommodation and age. Further in-depth research is needed to address these issues^14,17,21^.

This study has several limitations: The primary limitation is that the relatively small sample size and short-term follow-up. Methodologically, Vector analysis parameters are mathematically derived rather than directly measured, requiring cautious clinical interpretation. Additionally, Current international standards lack consensus on quantitative thresholds for distinguishing corneal versus internal astigmatism components. Our study primarily focused on the impact of ORA-ACA axial alignment consistency on outcomes. Further investigation into the influence of ORA axis classification types would be valuable for future research. With regard to population, The exclusive focus on myopic astigmatism excludes hyperopic, mixed astigmatism, and emmetropic eyes, with underrepresentation of adolescents and older adults, limiting generalizability. Confounding factors such as age, pupillary dynamics and crystalline lens accommodation changes should be considered.

## Conclusion

In WFG FS-LASIK surgery based on total ocular design, the consistency of axis between ORA and ACA affects the degree of deviation in astigmatic axis correction. However, this consistency does not have a significant impact on postoperative UDVA. High preoperative |ORA|/|RA| can lead to overcorrection of astigmatism, which suggests that astigmatism correction should be approached from a total eye perspective. RA and ACA are predominantly WTR, while ORA is predominantly ATR. Whether ORA is a constant value and the correlation between ORA and ACA or RA require further research with larger sample sizes to gain deeper reaserch.

## Data Availability

Datasets used and /or analyzed during the current study available from the corresponding author on reasonable request.
